# The effects of external knowledge source heterogeneity on enterprise process and product innovation performance

**DOI:** 10.1371/journal.pone.0234649

**Published:** 2020-06-12

**Authors:** Yuefang Si, Wanxin Liu, Xianzhong Cao

**Affiliations:** 1 The Center for Modern Chinese City Studies, East China Normal University, Shanghai, China; 2 School of Urban & Regional Science, East China Normal University, Shanghai, China; Institute of Geographic Sciences and Natural Resources Research (IGSNRR), Chinese Academy of Sciences (CAS), CHINA

## Abstract

As a global manufacturing centre, China is transitioning from a ‘Made in China’ to ‘Create in China’ perspective. An ever-increasing number of companies are developing new competitive advantages and improving their innovation levels by acquiring external knowledge. Yet, studies rarely discuss the influence of various sources of knowledge on process and product innovation performance in China’s manufacturing enterprises. Based on the Pavitt industry classification, we use a bivariate Probit model to investigate the influence of external knowledge sources on innovation performance, and test it by using Enterprise Survey data for China, published by the World Bank in 2013. Our empirical analysis indicates that external sources of knowledge, with the exception of suppliers, have a significantly positive influence on process and product innovation performance. Specifically, in the process of technological innovation, peers have a positive effect on enterprise process improvement, especially in the science-based sector. In product innovation, close technical cooperation with users accelerates the commercial manifestation of products, especially in the specialized supplier sector.

## 1 Introduction

How enterprises in China, a manufacturing powerhouse, upgrade their innovation ability has been a flashpoint in regional economics and business management research. The key to innovation lies in the acquisition, integration and transformation of different knowledge sources and related knowledge, knowledge with different attributes and their communication methods directly affect knowledge production and firm innovation performance [1]. In this era of open economies, while increasing their research and development (hereinafter, R&D) investment, businesses are also engaging in innovative activities by acquiring external sources of knowledge such as universities, research institutions, suppliers, users and competitors [2].

As early as 1973, Rothwell et al., in their study on how the British enterprise evolved via innovation after World War II, found that myriads of creative ideas were not sprouted inside firms, but rather, outside of them [3]. Thus, the concept of external knowledge as a source of innovation emerged. Subsequently, external sources of knowledge were introduced into the research of enterprise innovation performance. As domestic and foreign scholars continue probing into the innovation performance of companies, the research scope of external sources of knowledge has expanded from single to multi-dimensional, gradually shifting from identifying only product users as innovators to discovering innovators within suppliers, peers, universities and research institutions [4–6]. Existing research on external sources of knowledge focuses on exploring their relationship to enterprise innovation performance, and mostly considers these external sources to be positively related to enterprise innovation performance, and knowledge characteristics, acquisition and exchange have great influence on the development of enterprise innovation [7]; Chesbrough points out companies should take the initiative to explore the external knowledge landscape and effectively supplement their internal R&D environments with these innovative resources in order to enhance their capabilities [8]; Wang et al., after surveying 150 companies through empirical research, conclude that innovative information obtained from outside the enterprise is conducive in elevating the enterprise’s absorption capacity and can support its technological R&D [9]. However, Levinthal holds that too many sources of knowledge can generate cognitive barriers, cripple the ability of businesses to deploy technology and, thus, become a stumbling block to the progress of innovation performance [10]. Chen et al. argue that if the technology provided by the external source exceeds what the enterprise can handle, the excessive knowledge load will have a negative impact on innovation [11]. On this basis, others point out an inverted U-shaped relationship between external sources of knowledge and enterprise innovation performance [12].

Suppliers, for their crucial roles in process and product innovation in manufacturing companies, act as essential external sources of knowledge that drive companies to implement technological innovation [13]. Upstream suppliers provide enterprises with unique ideas, as well as new technologies by way of materials, parts or complete machines, in a way that mobilizes their innovation activities. Although core suppliers are familiar with the technology, process and management standards within the industry, a knowledge reserve similar to that of the enterprise itself, through cooperation with pioneering technology suppliers with innovation awareness, enterprises can quickly acquire market information and leading technical resources, so as to improve the quality of products and services, hence lifting their innovation capabilities [14].

Technological innovation is an ongoing process in which continuous improvement and product renewal are of paramount significance in transforming and upgrading Chinese manufacturing companies. Furthermore, Schumpeter’s innovation theory and its subsequent derivative theory suggest there are tremendous differences in the technical characteristics of different industrial enterprises [15]. Wang et al. believe the specific technology trajectory of a enterprise is pivotal in raising its innovation performance [9]. Scholars such as Li find clear synergy between process and product innovation, and consider this synergy the basis for realizing enterprise technology innovation [16]. However, few studies put process and product innovation under the same framework in order to analyse the impact of external sources of knowledge on them. In domestic research, studies rarely focus on the impact of external sources of knowledge on innovation performance of enterprises under different industrial and technological categories. In view of this, by leveraging the industry classification method proposed by Pavitt, an innovation economist in the UK, we explore the impact of a heterogenous cohort of external knowledge sources on enterprise process and product innovation, aiming to answer the following research questions: How do external sources of knowledge impact process and product innovation performance of Chinese companies overall and for specific industrial sectors? How can Chinese companies utilize these external sources of knowledge to best improve their process and product innovation performance? What are their differences?

The rest of the article is organized as follows. Section 2 presents a literature review of the external sources of knowledge and enterprise innovation performance, industry heterogeneity and theoretical hypothesis. The data collection and sample selection, the different variables, and the model specifications are detailed in Section 3. In Section 4, we present the main empirical results. Section 6 concludes and discusses implications for further research and policy.

## 2 Theoretical hypothesis

### 2.1 External sources of knowledge and enterprise innovation performance

As early as the 20th century, studies have shown that users are the most critical external source of knowledge for companies. Ren et al., through the analysis of successful domestic businesses, point out for technology-based companies, consumer demand from users is the primary knowledge source [17]. To successfully launch a new product or service, a company must accurately meet customer needs. Traditional market research can only obtain superficial information regarding user needs, however, Zhang et al. show if users are empowered to directly design or improve products, and if they can identify the function and appearance of the new product and the production level of the new process, this not only promotes the company’s enthusiasm for improving products or adding new features, but also helps the company meet consumer demand [18]. Therefore, many companies have placed R&D institutions at sites near consumers in order to understand the specific needs of their users and boost innovation.

Due to certain similarities in product innovation, production process design, management systems, and marketing strategies among peer companies, they can have interdependent and symbiotic relationship [19]. Relevant research indicates that peer knowledge sources are a crucial means for companies to bolster their innovation performance [11]. By following the innovative developments of peers, companies can acquire essential technical resources by means of technology licensing, technology acquisition, mergers and acquisitions and venture investments. Moreover, cooperation mechanisms amongst peers make it easier for companies to form complementary technological alliances. In addition, the natural competitive relationship can also inspire innovative ideas and stimulate innovation behaviours.

Finally, the development of key technologies by enterprises is also inseparable from the basic knowledge and application technology provided by universities and research institutions. Through cooperation with universities and research institutions companies can easily acquire advanced knowledge and technology while reducing R&D costs and innovation risks. Additionally, the vast talent pool of scientific and technical personnel in universities and research institutes guarantees the supply of high calibre, cutting-edge research. Furthermore, since universities and research institutions are not direct competitors of these companies, there is no dispute due to financial interest and cooperation can be maximized. As such, universities and research institutions are a sound source of knowledge for companies to implement process and product innovations.

This paper proposed the following hypotheses:

H1a: Cooperation with upstream suppliers has a positive impact on enterprise process and product innovation performance.

H1b: User feedback has a positive impact on enterprise process and product innovation performance.

H1c: Exchanges and collaborations with peers have a positive impact on enterprise process and product innovation performance.

H1d: Knowledge and technology from universities and research institutions have a positive impact on enterprise process and product innovation performance.

### 2.2 Industry heterogeneity

Pavitt’s industry classification standards have become a paradigm for scholars studying enterprise innovation [20]. Building upon this classification, we have sorted out various industrial sectors in China’s manufacturing industry based on enterprise characteristics, innovation sources, technical characteristics and innovation types. See Table 1 for details.

**Table 1:** Enterprise innovation characteristics based on Pavitt industry classification.

Category of industrial sectors	Business scale	Enterprise R&D capabilities	Source of technological innovation	Technical characteristics	Type of technological innovation
Supplier-led sector	Small	Relatively weak	Supplier	Carry out professional technical training to reduce process production costs	Process innovation
Scale-intensive sector	Large	Average	Peer	Improve large-scale production lines and enhance the ability of complex assembly systems	Process innovation
Specialized-supplier sector	Small	Relatively strong	User	Continuously improve product design and performance;	Product Innovation
				Rapid response to users’ needs	
Science-based sector	Large	Strong	University and research institutions	Technological innovation with a wide range of applications	Process / Product Innovation

Classifying Chinese manufacturing enterprises clarifies the industrial differentiation of external sources of knowledge on innovation performance and optimizes the search strategy for these sources. Specifically, the representative industries of the supplier-led sector include traditional manufacturing industries such as textiles, paper products and food processing. The companies in this sector are generally small in scale and weak in internal R&D and innovation capabilities. Their innovation resources of knowledge and technology largely come from equipment and raw material suppliers upstream in the industrial chain. Since buyers in this sector are comparatively sensitive to price, the R&D investment of these companies mainly focuses on professional skills training for workers and production cost reduction through process innovation. Li et al. conducted an empirical analysis of 221 enterprises in China’s textile industry, revealing that differences in dependence on suppliers cause differences in technological and product innovation performance [16]. The frequent technical exchanges and cooperation between companies and suppliers can be advantageous to enterprise innovation performance. Xu et al. find that the technological level of equipment and raw materials are the primary factors affecting innovation in China’s paper products industry [21]. This implies that the adoption of upstream suppliers’ technical knowledge is an efficacious path for these enterprises to innovate.

Enterprises in the scale-intensive sector are mainly engaged in the production of basic materials and durable consumer goods, such as metal products and transportation equipment manufacturing. The enterprises in this sector are usually large in scale, with a focus on technological innovation of processes and little interest in creating new products. Most of their innovation aims at upgrading production lines and improving complex assembly systems, thereby reducing production costs. The technology sources in this sector are mainly peer enterprises. In an environment where technological capabilities of enterprises is more or less the same, competitors can turn out to be innovation partners. By establishing industry standards with peers that can be widely applied, enterprises avoid incompatibilities by sharing technologies. This effectively cuts down unnecessary waste of resources, enabling enterprises to invest more in core R&D, thus enhancing the level of innovation.

The representative industry of the specialized-supplier sector is mainly instrumentation manufacturing. Being professional suppliers offering products for targeted consumers, such companies are often small in scale. However, because the enterprises in this sector provide various professional technical equipment tailored for users, and deliver specialized technical knowledge and operational experience, their R&D costs are generally high, as are their product prices. Ren et al., in elaborating on the innovation driven system of the specialized equipment manufacturing industry, find enterprises will set up an effective customer communication mechanism and obtain timely feedback on quality at each stage of R&D [22]. Enterprise technology innovation in such a sector has a strong reliance on users, and the company’s technology trajectory is more inclined towards product innovation that can elevate performance instead of trying to reduce costs through process innovation. For companies in this sector, the continuous perfection of product design and performance, and the ability to respond quickly to user needs is the key to success in innovation.

Finally, the science-based sector is typified by the electronic equipment manufacturing and chemical industries. With the rapid development of related sciences, universities and research institutions occupy a unique and major role in science and technology innovation. The basic knowledge and applied technology provided by science institutions is the chief source for companies in the science-based sector. Liu et al., through empirical research on the data of 12 major industrial sectors in China from 1950 to 1989, find that innovation activities in the science-based sector are mainly concerned with applying technical achievements of R&D laboratories to production [23]. Due to the wide applicability of science-based knowledge, the enterprises in this sector affect both process and product innovation.

In summary, our hypothesis put forward is as follows:

H2: There are industrial differences in the impact of external sources of knowledge on enterprise process and product innovation performance.

**Fig 1:**
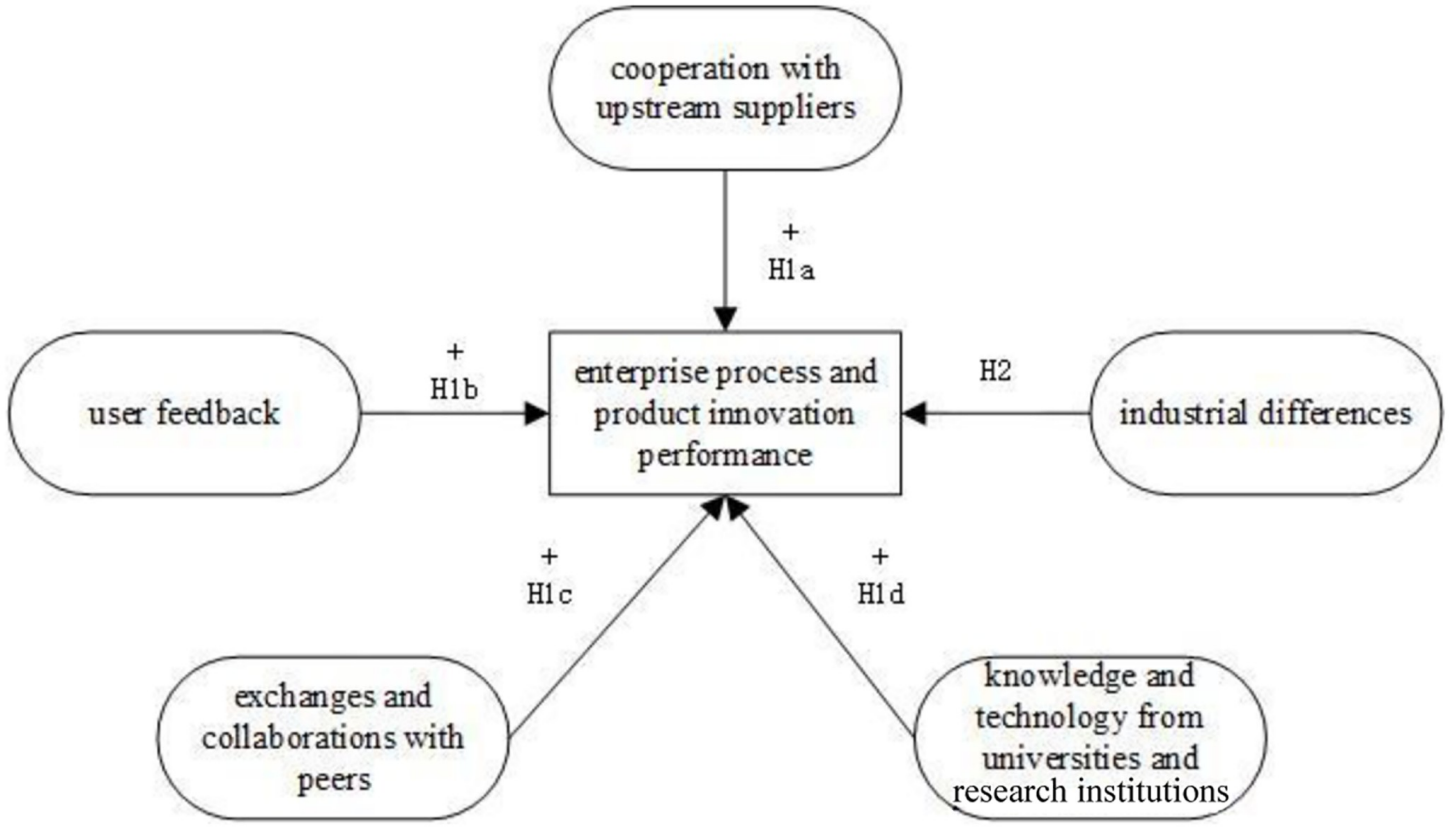
The relationship and analysis framework of theoretical hypothesis.

The relationship and analysis framework of theoretical hypothesis are shown in Fig 1.

## 3 Research design

### 3.1 Data source and sample selection

Taking the latest Enterprise Surveys from the World Bank as the database (https://data.worldbank.org), and Chinese manufacturing enterprises as research objects, we analyse the relationship between external sources of knowledge and enterprise innovation performance. The surveys from the World Bank are the result of collecting statistics from upper management, including CEOs and executives, and used a stratified random sampling. The three levels of stratification were industry type, enterprise size and geographical region. Its reliability and validity are high. The data covers 2,700 private enterprises and 148 state-owned enterprises in 23 cities including Beijing, Chengdu, Dalian, Dongguan, Foshan, Guangzhou, Hangzhou, Hefei, Jinan, Luoyang, Nanjing, Nantong, Qingdao, Shenyang, Shenzhen, Shijiazhuang, Suzhou, Tangshan, Wenzhou, Wuhan, Wuxi, Yantai and Zhengzhou. Considering the research subjects of this study were Chinese manufacturing companies, we screened out state-owned enterprises that did not specify their industry, as well as service and retail enterprises. As a result, we obtained cross-sectional data covering 1689 manufacturing enterprises in 15 industries, including textile, papermaking and paper products, wood processing and bamboo, rattan, palm and straw products industry, metal products industry, transportation equipment manufacturing industry, rubber and plastic products industry, nonmetallic mineral products industry, food and beverage manufacturing industry, metallurgy and metal rolling industry, electronic equipment manufacturing, general and specialized equipment manufacturing, textile and garment industry, fur and its products, instrumentation manufacturing (medical, precision optical instruments, watchmaking) industry, Chemical raw materials and chemical manufacturing, petroleum processing, cooking and nuclear fuel processing industry.

Among them, 79.8% adopted at least one type of technological innovation; 76.9% adopted a process innovation, 70.7% carried out a product innovation; 1141 out of the 1689 used both innovation methods, accounting for 67.5% of the total number. Based on the national economic industry classification criteria, we classified the industries to which the 1689 companies belong by referring to the aforementioned Pavitt’s industry classification method. Among the sample enterprises, 32.6% belong to the supplier-led sector; 23.3% the scale-intensive sector; 25.6% the specialized-supplier sector; 18.5% science-based sector. See Table 2 for details.

**Table 2:** Industry classification of sampled enterprises.

Sector category	Industry type	Quantity of enterprises
Supplier-led sector	Textile, papermaking and paper products, wood processing and bamboo, rattan, palm and straw products industry	551
Scale-intensive sector	Metal products industry, transportation equipment manufacturing industry, rubber and plastic products industry Nonmetallic mineral products industry, food and beverage manufacturing industry, metallurgy and metal rolling industry	393
Specialized-supplier sector	Electronic equipment manufacturing, general and specialized equipment manufacturing, textile and garment industry, fur and its products, instrumentation manufacturing (medical, precision optical instruments, watchmaking) industry	433
Science-based sector	Chemical raw materials and chemical manufacturing, petroleum processing, cooking and nuclear fuel processing industry	312

### 3.2 Variable definitions and measurement

Explained variables: Drawing lessons from the research findings of Bogliacino and Pianta [24], we divide innovation performance into two types: Process Innovation Performance (hereinafter, PC) and Product Innovation Performance (hereinafter, PD). Process innovation denotes the advancement in and optimization of production processes through new or substantially ameliorated production methods; product innovation refers to adding new functions to existing products or issuing new products [11]. PC is measured via three components; (1) introduction of new technologies and equipment to improve processes; (2) introduction of new quality control procedures in production and operation; and (3) adaptative behaviour to advance production flexibility. PD is also measured via three components; (1) introduction of new technologies and equipment to improve products; (2) introduction of new products; and (3) addition of new functions to existing products. A value of 1 is assigned to affirmative responses for both PC and PD measures.

Explanatory variables: This study applies four external sources of knowledge to both process and product innovation, resulting in a total of eight explanatory variables. These are suppliers, users, peers, and universities or research institutions.

Control variables: In line with previous literature on enterprise innovation performance, we have selected R&D investment, business age, and enterprise size as control variables [10]. All the variable are detailed in Table 3.

**Table 3:** Design of variables.

Variable type	Variable name	Variable measures
Dependent variables	Process Innovation Performance (PC)	Introduction of new technologies and equipment to improve processes
		Introduction of new quality control procedures in production and operation
		Adaptive behaviour to advance production flexibility
	Product Innovation Performance (PD)	Introduction of new technologies and equipment to improve products
		Introduction of new products
		Addition of new functions to existing products
Independent variables	Supplier-based Knowledge Source for Process Improvement (SUP_PC)	Cooperation and development with suppliers for process innovation
	User-based Knowledge Source for Process Improvement (USER_PC)	Cooperation with customers for process innovation
	Peer-based Knowledge Source for Process Improvement (PEER_PC)	Sharing of technical knowledge from and with peer companies for process innovation
	University & Research Institute-based Knowledge Source for Process Improvement (UNIV_PC)	Acquiring creative ideas from universities and research institutions for process innovation
	Supplier-based Knowledge Source for Product Improvement (SUP_PD)	Cooperation and development with suppliers for product innovation
	User-based Knowledge Source for Product Improvement (USER_PD)	Cooperation with customers for product innovation
	Peer-based Knowledge Source for Product Improvement (PEER_PD)	Launch products based on product information from peer companies
	University & Research Institute-based Knowledge Source for Product Improvement (UNIV_PD)	Acquiring creative ideas from universities and research institutions for product innovation
Control variables	R&D investment (RD)	Is there an R&D investment?
	Business age (AGE)	Duration since establishment—take the natural logarithm
	Enterprise size (SIZE)	Quantity of staff—take the natural logarithm

### 3.3 Model formulation

Given that there is often a synergy between technological improvement and new product creation in technological innovation, the stochastic disturbance terms, ε_1_ and ε_2_, might be correlated [16]. If we use a univariate Probit model for the two explanatory variables PC and PD, the efficiency might be lost [25]. Therefore, this study adopts a non-censored bivariate Probit model for simultaneous estimation, so as to control correlation between random error terms. The basic formula of the bivariate Probit model is as follows:

$$\text{Prob}(Y=1)=\int_{-\infty }^{\beta x}\Phi (t)dt=\frac{e^{\beta x}}{1+e^{\beta x}}$$ (1)

The specific model is as follows:

$$\{\begin{array}{l} Y_{A}=a+\beta_{1}SUP_{PC}+\beta_{2}USER_{PC}+\beta_{3}PEER_{PC}+\beta_{4}UNVI_{PC}+\beta_{5}RD+\beta_{6}AGE+\beta_{7}SIZE+\varepsilon_{1}\\ Y_{B}=b+\delta_{1}SUP_{PC}+\delta_{2}USER_{PC}+\delta_{3}PEER_{PC}+\delta_{4}UNVI_{PC}+\delta_{5}RD+\delta_{6}AGE+\delta_{7}SIZE+\varepsilon_{2} \end{array}$$ (2)

In formula (2), *Y*_*A*_ and *Y*_*B*_ represent PC and PD, respectively; *a* and *b* are constants; β denotes the corresponding estimated coefficient; the stochastic disturbance terms (*ε*_*1*_, *ε*_*2*_) comply with a two-dimensional joint normal distribution with the expectation value of 0 and variance of 1. They follow the equations as shown below:

$$\{\begin{array}{c} E(\varepsilon_{1})=E(\varepsilon_{2})=0\\ var(\varepsilon_{1})=var(\varepsilon_{2})=1\\ cov(\varepsilon_{1},\varepsilon_{2})=\rho \end{array}$$ (3)

## 4 Empirical analysis

From the matrix of correlation coefficients for each variable, the correlation coefficients between variables are all 0.5 or less, thus we conclude the multicollinearity problem does not exist.

Table 4 demonstrates the regression results of the impact of external sources of knowledge on enterprise innovation performance. Models (1)-(5) represent the regression results of manufacturing enterprises as a whole, the supplier-led sector, the scale-intensive sector, the specialized-supplier sector and the science-based sector respectively. The Wald test values of models (1)-(5) are 259.90, 99.06, 103.90, 92.55 and 62.07, respectively, all of which are at a 1% level of significance. This indicates that there is a relationship between enterprise process and product innovation activities and external sources of knowledge, the results of which are proven with reliability and validity by the bivariate Probit model.

**Table 4:** Bivariate Probit model regression results.

	Manufacturing enterprises as a whole		Supplier-led sector		Scale-intensive sector		Specialized-supplier sector		Science-based sector	
	(1)		(2)		(3)		(4)		(5)	
	PC	PD	PC	PD	PC	PD	PC	PD	PC	PD
SUP	-0.274***	0.007	-0.341*	-0.042	-0.285	-0.180	-0.452**	-0.079	0.098	0.267
	(0.103)	(0.130)	(0.186)	(0.265)	(0.210)	(0.255)	(0.186)	(0.2239)	(0.281)	(0.294)
USER	0.253**	0.617***	0.238	0.699***	0.217	0.840***	0.365*	0.594***	0.113	0.459*
	(0.114)	(0.119)	(0.200)	(0.239)	(0.241)	(0.264)	(0.201)	(0.1848)	(0.288)	(0.260)
PEER	0.665***	0.811***	0.259	1.189***	1.535***	1.297***	0.778***	0.466**	0.622**	0.309
	(0.128)	(0.108)	(0.193)	(0.195)	(0.441)	(0.285)	(0.281)	(0.191)	(0.300)	(0.231)
UNIV	0.544***	0.277**	0.771***	0.400*	0.768***	0.170	0.593**	0.641***	0.068	0.021
	(0.135)	(0.121)	(0.239)	(0.204)	(0.293)	(0.285)	(0.270)	(0.225)	(0.305)	(0.268)
1RD	0.766***	1.054***	5.243***	0.074***	0.633***	1.440***	0.888***	0.824***	1.479***	1.049***
	(0.103)	(0.103)	(0.138)	(0.349)	(0.220)	(0.675)	(0.196)	(0.184)	(0.293)	(0.218)
AGE	0.121	-0.043	0.051	0.062	-0.118	0.236	0.240	-0.167	0.368*	-0.324*
	(0.091)	(0.082)	(0.067)	(0.063)	(0.175)	(0.164)	(0.168)	(0.158)	(0.215)	(0.181)
SIZE	-0.004	0.000	0.344	-0.194	-0.003	-0.106	-0.027	-0.053	-0.069	0.103
	(0.039)	(0.034)	(0.438)	(0.429)	(0.471)	(0.072)	(0.075)	(0.067)	(0.095)	(0.078)
Constant	0.295	0.077	0.370	-0.185	0.797	-0.161	-0.002	0.675	-0.110	0.528
	(0.249)	(0.233)	(0.442)	(0.428)	(0.469)	0.444	(0.509)	(0.500)	(0.627)	(0.508)
Wald chi	259.50***		99.06***		103.90***		92.55***		62.07***	
	(0.000)		(0.000)		(0.000)		(0.000)		(0.000)	
N	1409		451		331		359		268	

① Take process innovation performance (PC) as the dependent variable, and the process knowledge source (SUP_PC, USER_PC, PEER_PC, UNIV_PC) as the independent variables; take the product innovation performance (PD) as the dependent variable, and the product knowledge source (SUP_PD, USER_PD, PEER_PD, UNIV_PD) as the independent variables).②***, **, * are indicated at the sig. level of 1%, 5%, and 10% respectively; the standard deviations are in brackets, the same below.

Model (1) in Table 4 lists the empirical results based on a sample of all manufacturing enterprises. The regression coefficients of users (0.253, 5% significance for PC; 0.617, 1% significance for PD), peers (0.665, 1% significance for PC; 0.811, 1% significance for PD), universities and scientific research institutions (0.544, 1% significance for PC; 0.277, 5% significance for PD) are all significantly positive, indicating these knowledge sources all promote enterprise PC and PD, hence verifying hypotheses 1b, 1c, and 1d. Hypothesis 1a, however, is not verified because although the regression coefficient of supplier for PC is -0.274 (1% significance), for PD it is 0.007, which is not significant. This might be explained by the fact that there are multiple problems in suppliers providing materials and equipment in China’s manufacturing industry, such as inadequate knowledge reserves, unqualified post-sales service and support ability and insufficient confidentiality awareness, which pose difficulties in backing enterprises’ attempts in process and product innovation. As a result, the correlation between suppliers and enterprise innovation performance is weak at best and, in fact, has a negative impact on enterprise process innovation.

Through analysing model (2), we find that in the supplier-led sector, the regression coefficient of suppliers on enterprise PC is -0.341 (10% significance), implying that its impact is weak; the regression coefficient of suppliers on enterprise PD is -0.042, which is not significant; The regression coefficient of users on enterprise PD is 0.699 (1% significance), implying that its impact is strong, the regression coefficient of suppliers on enterprise PC is 0.238, which is not significant; The regression coefficient of peers on enterprise PD is 1.189 (1% significance), implying that its impact is strong, the regression coefficient of suppliers on enterprise PC is 0.259, which is not significant; The regression coefficient of universities and scientific research institutions on enterprise PC is 0.771 (1% significance), implying that its impact is strong, the regression coefficient of suppliers on enterprise PD is 0.400 (10% significance), which is weak. Our conclusion here is similar, although this sector relies on upstream suppliers, due to the weak R&D capabilities of upstream suppliers in China’s manufacturing industry, the outdated production process and the single product variety, the enterprises in this sector often find it hard to upgrade their innovation level by cooperating with suppliers.

From model (3) we can see that in the scale-intensive sector, the regression coefficient of suppliers on enterprise PC and PD is -0.285 and -0.180, which are both not significant; The regression coefficient of users on enterprise PD is 0.840 (1% significance), implying that its impact is strong, the regression coefficient of suppliers on enterprise PC is 0.217, which is not significant; The regression coefficient of universities and scientific research institutions on enterprise PC is 0.768 (1% significance), implying that its impact is strong, the regression coefficient of suppliers on enterprise PD is 0.170, which is weak. When a company conducts process and product innovation, the influence factor of peer companies on PC and PD is 1.535 and 1.297 respectively (both at 1% significance). This shows that technical help and innovative information from companies in the industry not only contribute to process technology improvement, but also help companies innovate products. There is a certain technical similarity among peers. Sharing of technical resources are an effective way for enterprises to rapidly improve their innovation capabilities. In addition, joint research with complementary peers help shorten the development cycle, reduce product development risks and ultimately accelerate the commercialization of R&D results.

Through model (4) it is found that in the specialized-supplier sector, the regression coefficient of suppliers on enterprise PC is -0.452 (5% significance), implying that its impact is weak; the regression coefficient of suppliers on enterprise PD is -0.079, which is not significant; The regression coefficient of peers on enterprise PC is 0.788 (1% significance), implying that its impact is strong, the regression coefficient of suppliers on enterprise PD is 0.466 (5% significance), which is weak; The regression coefficient of universities and scientific research institutions on enterprise PC is 0.593 (5% significance), implying that its impact is weak, the regression coefficient of suppliers on enterprise PD is 0.641 (1% significance), which is strong. The regression coefficients of the user as a knowledge source are 0.365 (10% significance) on PC and 0.594 (1% significance) on PD indicating a positive correlation to the company’s innovation performance, particularly in PD. This suggests that the creative ideas and external market information provided by users can enable enterprises to grasp market demand in a “targeted, fast and resolute” manner and reduce R&D risks. Through face-to-face communication with customers, who ultimately are the end-users of any new products or processes resulting in new or improved products, companies can continuously improve process technology and product performance, thus enhancing their ability to innovate.

Finally, model (5) demonstrates that in the science-based sector, the regression coefficient of suppliers on enterprise PC and PD is 0.098 and 0.267, which are both not significant; The regression coefficient of users on enterprise PD is 0.459 (10% significance), implying that its impact is weak, the regression coefficient of suppliers on enterprise PC is 0.113, which is not significant; The regression coefficient of peers on enterprise PC is 0.622 (5% significance), implying that its impact is weak, the regression coefficient of suppliers on enterprise PD is 0.309, which is not significant. Universities and research institutions have impact coefficients of 0.068 and 0.021 on PC and PD, respectively, which are not significant. In general, universities and research institutions are crucial sources of external knowledge, and many companies rely heavily on their cutting-edge scientific and technological research in innovation. In the theory of regional innovation system, universities and research institutions play the role of knowledge producers, while enterprises play the role of knowledge transformation [26]. However, whether companies can achieve technological innovation using this information depends on their ability to learn and internalize it. We are reminded of the findings of Levinthal [10] that and excess of information, beyond what a enterprise can handle, results in a negative effect on innovation.

According to our analysis of models (1)-(5), we find that the influence of external sources of knowledge on PC and PD does differ among industries, thus hypothesis 2 is verified. The main reason is that different types of industries have different needs for innovation. The innovation needs of specialized-supplier sector come from users’ feedback information, the innovation needs of scale-intensive sector come from peer exchanges and cooperation, and the innovation needs of science-based sector come from knowledge cooperation of universities and scientific research institutions. As seen above, feedback from users is essential for creating new products by the specialized-supplier sector (0.594 at 1% significance) and exchanges with peers for companies in the scale-intensive sector is of paramount importance (1.535 PC and 1.297 PD at 1% significance). Although not statistically significant, the cutting-edge research provided by universities and research institutions can serve as an important gateway of innovation for manufacturing enterprises.

## 5 Conclusion and discussion

Studying the impact of various external sources of knowledge on manufacturing enterprise innovation provides a reference for companies to rationally choose appropriate sources, which, in turn, contributes to improving innovation management of China’s manufacturing industry at the micro-level. Based on data from the 2013 World Bank Enterprise Survey for China, we see the impact of different external sources of knowledge on enterprise innovation performance, and the differences in impact among various sectors. According to our findings, when choosing suppliers, supplier-led enterprises should place importance on the innovative characteristics of suppliers and whether they can effectively apply their innovation resources. Secondly, companies in the scale-intensive sector should focus on obtaining and implementing technical information from peers in a timely manner and mastering the technological trends of the industry as a way to reduce innovation costs generated from blind technology searches. Thirdly, enterprises in the specialized-supplier sector can embrace the user innovation model to gain market share, accelerate the building of core competencies and maintain a leading position in an industry with fierce competition. Finally, universities and research institutions should target cutting-edge technology and original research achievements with significant real-world application value. Similarly, internal innovation centres for companies should focus on process improvement and product creation in the medium to low-end, practical market. Enterprises in the science-based sector need to be flexible when cooperating with universities and research institutions, and attune their R&D to market demand.

Compared with the existing research, the research contribution of this paper lies in the analysis of manufacturing enterprises process and product innovation performance, and finds that three types of external knowledge sources, namely users, peers, universities and scientific research institutions, have significant positive promoting effects, and in-depth analysis is made from different industries, which is less concerned in previous research. Based on the above analysis, the following suggestions are proposed: First, China’s manufacturing enterprises should take initiatives in expanding the scope of external sources of knowledge and encourage the acquisition of diverse knowledge and skills. Second, as the primary innovators in the market, enterprises play a decisive role in transforming creative ideas into actual processes and products. Therefore, enterprises should actively strengthen their capacity in incorporating knowledge, including precise acquisition, timely comprehension, efficient transformation and utilization of external knowledge in order to achieve technological transformation and advancement.

In the future, firstly, external sources of knowledge can be further diversified to include consultants, governments, and foreign agencies to name a few. Different sources will vary in their purpose and method regarding innovation, and their influences will also be different. Therefore, investigations of additional external sources that might influence innovation performance can be undertaken to expand this research. Secondly, a comparative study could be conducted on the heterogeneity of regions with regard to external sources of knowledge, which can lead to a path towards enterprise knowledge sources that are even more useful in practice.

## Supporting information

S1 Data (ZIP)
