# Supplementary material for: The effects of external knowledge source heterogeneity on enterprise process and product innovation performance
Source: PLoS One. 2020 Jun 12;15(6):e0234649. doi: 10.1371/journal.pone.0234649 (PMC7292382; doi:10.1371/journal.pone.0234649)
Supplement: S1 Data — (ZIP) [file pone.0234649.s001.zip › data/China ES_Manufacturing_English_FINAL.pdf]

SERIAL NUMBER [ ]

THE WORLD BANK

China - Enterprise Survey

Manufacturing Module (2012)

GPS Coordinates	[TO BE COMPLETED USING GOOGLE MAPS]
Degrees North (Latitude)	lat
Degrees East (Longitude)	lon

A. CONTROL INFORMATION [TO BE COMPLETED BEFORE INTERVIEW]

**A.0 Questionnaire** a0	**Module**
Manufacturing	1

A.1 Country

a1

A.1a Language

a1a

**A.2**	**Sampling Region** a2
Hefei City	1
Beijing (municipalities)	2
Guangzhou City	3
Shenzhen City	4
Foshan City	5
Dongguan City	6
Shijiazhuang City	7
Tangshan City	8
Zhengzhou City	9
Luoyang City	10
Wuhan City	11
Nanjing City	12
Wuxi City	13
Suzhou City	14
Nantong City	15
Shenyang City	16
Dalian City	17
Jinan City	18
Qingdao City	19
Yantai City	20
Shanghai (municipalities)	21
Chengdu City	22
Hangzhou City	23
Ningbo City	24
Wenzhou City	25

**A.3a**	**Screener Region** (coded ex post) a3a
Hefei City	1
Beijing (municipalities)	2
Guangzhou City	3
Shenzhen City	4
Foshan City	5
Dongguan City	6
Shijiazhuang City	7
Tangshan City	8
Zhengzhou City	9
Luoyang City	10
Wuhan City	11
Nanjing City	12
Wuxi City	13
Suzhou City	14
Nantong City	15
Shenyang City	16
Dalian City	17
Jinan City	18
Qingdao City	19
Yantai City	20
Shanghai (municipalities)	21
Chengdu City	22
Hangzhou City	23
Ningbo City	24
Wenzhou City	25

A.3x Name of city/town/village

a3x

A.3b Is this city the official capital city? a3b

Yes	1
No	2

A.3c Is this city the main business city? a3c

Yes	1
No	2

A.3 Size of locality **a3**

City with population over 1 million – other than capital	2
Over 250.000 to 1 million	3
50.000 to 250.000	4
Less than 50.000	5

A.4 Industry

		Sampling sector **a4a**	Screener sector **a4b**
**Manufacturing :Section D**	Food	15	15
	Tobacco	16	16
	Textiles	17	17
	Garments	18	18
	Leather	19	19
	Wood	20	20
	Paper	21	21
	Recorded media	22	22
	Refined petroleum product	23	23
	Chemicals	24	24
	Plastics & rubber	25	25
	Non metallic mineral products	26	26
	Basic metals	27	27
	Fabricated metal products	28	28
	Machinery and equipment	29	29
	Electronics (31 & 32)	31	31
	Precision instruments	33	33
	Transport machines (34&35)	34	34
	Furniture	36	36
	Recycling	37	37
**Government owned**	100% state owned enterprise	100	100
**Retail**	Retail	52	52
**Other Services**	Wholesale	51	51
	IT	72	72
	Hotel and restaurants: section H	55	55
	Services of motor vehicles	50	50
	Construction Section F:	45	45
	Transport Section I: (60-64)	60	60

A.5 Sector match between screener information and sample frame **a5**

Yes, screener and sample frame info match	1
No, screener and sample frame do not match but establishment still does activities which match sample frame	2
No, does not match	3

**A.6** Size	**Sampling size a6a**	**Screener Size a6b**
Small >=5 and <=19	1	1
Medium >=20 and <=99	2	2
Large >=100	3	3

A.7 Establishment is part of a larger firm **a7**

Yes	1
No, a firm on its own	2

A.7a

Number of establishments that form the firm	**a7a**

A.8 Type of establishment **a8**

HQ without production and/or sales in this location	1
HQ with production and/or sales in this location	2
Establishment physically separated from HQ and other establishments of the same firm	3
Establishment physically separated from HQ but with other establishments of the same firm	4
**Does not apply**	**-7**

A.9 Are establishment's financial statements prepared separately from HQ's statements**?** **a9**

Yes	1
No	2
**Does not apply**	**-7**

A.10 Are establishment's financial statements prepared separately from other establishments of the same firm? **a10**

Yes	1
No	2
**Does not apply**	**-7**

A.11 If HQ, are financial statements independent from the rest of establishments? **a11**

Yes	1
No	2
**Does not apply**	**-7**

A.12 Interviewer code **a12**

A.13 Supervisor code **a13**

SERIAL NUMBER | |

A.14 Time face-to-face interview begins:

Day (dd)	Month (mm)	Year (yyyy)	Hour (00 to 23)	Minutes (00 to 59)
a14d	a14m	a14y	a14h	a14min

READ THE FOLLOWING TO THE RESPONDENT BEFORE PROCEEDING.
The goal of this survey is to gather information and opinions about the business environment in China. The information gathered here will help the World Bank to develop new policies and programs that enhance employment and economic growth.

The information obtained here will be held in the strictest confidentiality. Neither your name nor the name of your business will be used in any document based on this survey.

B. GENERAL INFORMATION

READ OUT THE FOLLOWING INTRODUCTORY SENTENCE ONLY IF A7 = 1 (yes):
The first few questions apply to the firm which your establishment is part of.

**B.1**	What is this firm's current legal status? **SHOW CARD 1**

Shareholding company with shares traded in the stock market	1	
Shareholding company with non-traded shares or shares traded privately	2	
Sole proprietorship	3	***SKIP TO B.2***
Partnership	4	
Limited partnership	5	
**Other (spontaneous - specify)___b1x___**	6	
**Don't know (spontaneous)**	**-9**	
		**b1**

**B.3**	What percentage of this firm does the largest owner or owners own?

	**Percent**
Percentage held by largest owner or owners	**b3** %
**Don't know (spontaneous)**	**-9**

SERIAL NUMBER [ ]

**B.2**	What percentage of this firm is owned by each of the following: **SHOW CARD 2**

	Percent	Don't know (spontaneous)
Private domestic individuals, companies or organizations	b2a %	-9
Private foreign individuals, companies or organizations	b2b %	-9
Government or State	b2c %	-9
Other	b2d %	-9
	100%	

If 100% terminate interview UNLESS a4a is 100 (state owned)

INTERVIEWER: CHECK THAT TOTAL SUMS TO 100%
(UNLESS RESPONDENT DOES NOT KNOW)

**B.4**	Amongst the owners of the firm, are there any females?

Yes	1	
No	2	
**Don't know (spontaneous)**	**-9**	
		**b4**

READ ONLY IF A7=1 (yes)
I want to proceed by asking you about this establishment only.

**B.5**	In what year did this establishment begin operations?

	Year
Year establishment began operations	**b5**
**Don't know (spontaneous)**	**-9**

INTERVIEWER: PROVIDE FOUR DIGITS FOR YEAR

**B.6**	How many full-time employees did this establishment employ when it started operations? Please include all employees and managers **(INTERVIEWER: include respondent when applicable)**

	Number
Full-time employees at start-up	**b6**
**Don't know (spontaneous)**	**-9**

**B.6a**	Was this establishment formally registered when it began operations?

Yes	1	
No	2	
**Don't know (spontaneous)**	**-9**	
		**b6a**

**B.6b**	In what year was this establishment formally registered?

	**Year**
Year establishment formally registered	**b6b**
**Don't know (spontaneous)**	**-9**
**Never registered (spontaneous)**	**-7**

INTERVIEWER: PROVIDE FOUR DIGITS FOR YEAR.

**B.7**	How many years of experience working in this sector does the Top Manager have?

	**Years**
Manager's experience in sector	**b7**
**Less than one year**	**1**
**Don't know (spontaneous)**	**-9**

**B.7a**	Is the Top Manager female?

Yes	1	
No	2	
**Don't know (spontaneous)**	**-9**	
		**b7a**

**B.8**	Does this establishment have an internationally-recognized quality certification? **(INTERVIEWER: if there is need for clarification, some examples are: ISO 9000 or 14000, or HACCP)**

Yes	1	
No	2	
**Still in process**	**-6**	
**Don't know (spontaneous)**	**-9**	
		**b8**

SERIAL NUMBER | |

C. INFRASTRUCTURE AND SERVICES

READ THE FOLLOWING TO THE RESPONDENT BEFORE PROCEEDING.
Now, we turn to the establishment's operations in fiscal year 2011.

**C.3**	Over the last two years, did this establishment submit an application to obtain an electrical connection?

Yes	1	
No	2	*GO TO QUESTION C.6*
**Don't know (spontaneous)**	**-9**	*GO TO QUESTION C.6*
		**c3**

**C.4**	In reference to that application for an electrical connection, approximately how many days did it take to obtain it from the day of the application to the day the service was received?

	**Days**
Wait for electrical connection	**c4**
**Less than one day**	**1**
**Still in process**	**-6**
**Application denied**	**-5**
**Don't know (spontaneous)**	**-9**

**C.5**	In reference to that application for an electrical connection, was an informal gift or payment expected or requested?

Yes	1	
No	2	
**Don't know (spontaneous)**	**-9**	
**REFUSE (spontaneous)**	**-8**	
		**c5**

**C.6**	Over fiscal year **2011**, did this establishment experience power outages?

Yes	1	
No	2	*GO TO QUESTION C.10*
**Don't know (spontaneous)**	**-9**	*GO TO QUESTION C.10*
		**c6**

**C.7**	In a typical month, over fiscal year **2011**, how many power outages did this establishment experience?

	**Number**	
Average number of power outages per month	**c7**	*IF 0, GO TO QUESTION C.10*
**Don't know (spontaneous)**	**-9**	

**C.8**	How long did these power outages last on average?

	**Hours**
Average duration of power outages	**c8**
**Less than one hour**	**1**
**Don't know (spontaneous)**	**-9**

**C.9**	Please estimate the losses that resulted from power outages either as a percentage of total annual sales or as total annual losses.

	**Percent**
Loss as percentage of total annual sales due to power outages	**c9a** %
**None**	**0**
**Don't know (spontaneous)**	**-9**

PROVIDE EITHER ONE OR THE OTHER, NOT BOTH

	**Yuan**
Annual losses due to power outages	**c9b**
**None**	**0**
**Don't know (spontaneous)**	**-9**

**C.10**	Over the course of fiscal year **2011**, did this establishment own or share a generator?

Yes	1	
No	2	***GO TO QUESTION C.12***
**Don't know (spontaneous)**	**-9**	***GO TO QUESTION C.12***
	**c10**	

**C.11**	In fiscal year **2011**, what percentage of this establishment's electricity came from a generator or generators that the establishment owned or shared?

	**Percent**
Percentage electricity from generators	**c11** %
**Don't know (spontaneous)**	**-9**

**C.12**	Over the last two years, did this establishment submit an application to obtain a water connection?

Yes	1	
No	2	***GO TO QUESTION C.15***
**Don't know**	**-9**	***GO TO QUESTION C.15***
	**c12**	

SERIAL NUMBER [ ]

**C.13**	In reference to that application for a water connection, approximately how many days did it take to obtain it from the day of the application to the day the service was received?

	Days
Wait for water connection	c13
**Less than one day**	**1**
**Still in process**	**-6**
**Application denied**	**-5**
**Don't know (spontaneous)**	**-9**

**C.14**	In reference to that application for a water connection, was an informal gift or payment expected or requested?

Yes	1	
No	2	
**Don't know (spontaneous)**	**-9**	
**REFUSE (spontaneous)**	**-8**	
		c14

**C.15**	Over fiscal year **2011**, did this establishment experience insufficient water supply for production?

Yes	1	
No	2	*GO TO QUESTION C.18*
**Don't know (spontaneous)**	**-9**	*GO TO QUESTION C.18*
		c15

**C.16**	In a typical month, over fiscal year **2011**, how many incidents of insufficient water supply did this establishment experience?

	Number	
Average number of incidents of water insufficiency per month	c16	*IF 0, GO TO QUESTION C.18*
**Don't know (spontaneous)**	**-9**	

**C.17**	How long did these incidents of insufficient water supply last on average?

	Hours
Average duration of insufficient water supply	c17
**Less than one hour**	**1**
**Don't know (spontaneous)**	**-9**

**C.18**	In fiscal year **2011**, what percentage of this establishment's water supply, used in the production process, was from public sources?

	Percent
Water from public sources	c18 %
**None**	**0**
**Don't Know**	**-9**

SERIAL NUMBER | |

**C.19**	Over the last two years, did this establishment submit an application to obtain a telephone connection?

Yes	1	
No	2	*GO TO QUESTION C.22*
**Don't know (spontaneous)**	**-9**	*GO TO QUESTION C.22*
	**c19**	

**C.20**	In reference to that application for a telephone connection, approximately how many days did it take to obtain it from the day of the application to the day the service was received?

	**Days**
Wait for telephone connection	**c20**
**Less than one day**	**1**
**Still in process**	**-6**
**Application denied**	**-5**
**Don't know (spontaneous)**	**-9**

**C.21**	In reference to that application for a telephone connection, was an informal gift or payment expected or requested?

Yes	1	
No	2	
**Don't know (spontaneous)**	**-9**	
**REFUSE (spontaneous)**	**-8**	
		**c21**

**C.22a**	At the present time, does this establishment use e-mail to communicate with clients or suppliers?

Yes	1	
No	2	
**Don't know (spontaneous)**	**-9**	
	**c22a**	

**C.22b**	At the present time, does this establishment use its own website?

Yes	1	
No	2	
**Don't know (spontaneous)**	**-9**	
	**c22b**	

**C.23**	Does this establishment have a high-speed Internet connection on its premises?

Yes	1	
No	2	*GO TO QUESTION C.28*
**Don't know (spontaneous)**	**-9**	*GO TO QUESTION C.28*
	**c23**	

**C.24**	Is this establishment's Internet connection used to:

**INTERVIEWER: READ EACH OPTION ALOUD**		**Yes**	**No**	**Don't Know (spontaneous)**	**NA (spontaneous)**
Make purchases for this establishment	**c24b**	1	2	**-9**	**-7**
Sell and market products	**c24f**	1	2	**-9**	**-7**
Do research and develop ideas on new products and services	**c24d**	1	2	**-9**	**-7**

**C.28**	Does this establishment currently use cell phones for the operations of the establishment?

Yes	1
No	2
**Don't know (spontaneous)**	**-9**
	**c28**

**C.30**	Using the response options on the card; To what degree is **Electricity** an obstacle to the current operations of this establishment? **SHOW CARD 3**<br><br>Using the response options on the card; To what degree is **Telecommunications** an obstacle to the current operations of this establishment? **SHOW CARD 3**

		No obstacle	Minor obstacle	Moderate obstacle	Major obstacle	Very Severe Obstacle	**Don't Know (spontaneous)**	**Does Not Apply (spontaneous)**
Electricity	**c30a**	0	1	2	3	4	**-9**	**-7**
Telecommunications	**c30b**	0	1	2	3	4	**-9**	**-7**

SERIAL NUMBER | |

D. SALES AND SUPPLIES

READ THE FOLLOWING TO THE RESPONDENT BEFORE PROCEEDING:
The next topic to be covered is how and where this establishment makes its sales.

**D.1a1**	In fiscal year **2011**, what was this establishment's main product, that is, the product that represented the largest proportion of annual sales?

Description
d1a1x

**D.1a2**	**TO BE CODED AFTER THE INTERVIEW** please choose the 4-digit code that best applies to the main product of this establishment.

	Code
Code of main product	d1a2
**Don't know (spontaneous)**	**-9**

**D.1a3**	What percentage of total sales does the main product represent?

	Percent
Percentage of sales represented by main product	d1a3 %
**Don't know (spontaneous)**	**-9**

**D.2**	In fiscal year **2011**, what were this establishment's total annual sales for ALL products and services?

	Yuan
Last complete fiscal year's total sales	d2
**Don't know (spontaneous)**	**-9**
**Please also write out the number (i.e. 50,000 as Fifty Thousand)**	
d2x	

**N.3**	In fiscal year **2009,** three fiscal years ago, what were total annual sales for this establishment?

	Yuan
Total annual sales three years ago	n3
**If establishment was not in business three years ago**	**-7**
**Don't know (spontaneous)**	**-9**

SERIAL NUMBER | |

D.3	In fiscal year **2011**, what percentage of this establishment's sales were:<br>**(INTERVIEWER: these must be asked in the order they appear on the table. The skip patterns must be followed in the oder they appear.)**<br>**SHOW CARD 5**

	**Percent**	**Don't know (spontaneous)**	
National sales	**d3a** %	**-9**	***IF 100, GO TO QUESTION D.10***
Indirect exports (sold domestically to third party that exports products)	**d3b** %	**-9**	***IF 100, GO TO QUESTION D.8***
Direct exports	**d3c** %	**-9**	***IF 0, GO TO QUESTION D.8***
	**100%**		

INTERVIEWER: CHECK THAT TOTAL SUMS TO 100%
(UNLESS RESPONDENT DOES NOT KNOW)

D.4	In fiscal year **2011**, when this establishment exported goods directly, how many days did it take on average from the time this establishment's goods arrived at their main point of exit (e.g., port, airport) until the time these goods cleared customs?

	**Days**
Average number of days to clear customs	**d4**
**Less than one day**	**1**
**Don't know (spontaneous)**	**-9**

D.6	In fiscal year **2011**, what percentage of the value of the products exported directly was lost while in transit because of theft?

	**Percent**
Losses due to theft as percentage of the value of the products	**d6** %
**No losses**	**0**
**Don't know (spontaneous)**	**-9**

D.7	In fiscal year **2011**, what percentage of the value of the products exported directly was lost while in transit because of breakage or spoilage?

	**Percent**
Losses due to breakage or spoilage as percentage of the value of the products	**d7** %
**No losses**	**0**
**Don't know (spontaneous)**	**-9**

D.8	In which year did this establishment first export directly or indirectly?

	**Year**
Began exporting directly or indirectly	**d8**
**Don't know (spontaneous)**	**-9**

**D.10**	In fiscal year **2011**, what percentage of the value of products this establishment shipped to supply domestic markets was lost while in transit because of theft?

	**Percent**
Losses due to theft as percentage of the value of the products	d10 %
**No losses**	**0**
**Don't know (spontaneous)**	**-9**
**No internal shipments made**	**-7**

**D.11**	In fiscal year **2011**, what percentage of value of products this establishment shipped to supply domestic markets was lost while in transit because of breakage or spoilage?

	**Percent**
Losses due to breakage or spoilage as percentage of the value of the products	d11 %
**No losses**	**0**
**Don't know (spontaneous)**	**-9**
**No internal shipments made**	**-7**

**D.12**	In fiscal year **2011**, as a proportion of all material inputs or supplies purchased that year, what percentage of this establishment's material inputs or supplies were: **SHOW CARD 6**

	**Percent**	**Don't know (spontaneous)**	
Material inputs or supplies of domestic origin	d12a %	-9	
Material inputs or supplies of foreign origin	d12b %	-9	*IF 0, GO TO QUESTION D.16*
	**100%**		

INTERVIEWER: CHECK THAT TOTAL SUMS TO 100%
(UNLESS RESPONDENT DOES NOT KNOW)

**D.13**	Were any of the material inputs or supplies purchased in fiscal year **2011,** imported directly?

Yes	1	
No	2	*GO TO QUESTION D.16*
**Don't know (spontaneous)**	**-9**	*GO TO QUESTION D.16*
		d13

**D.14**	In fiscal year **2011**, when this establishment imported material inputs or supplies, how many days did it take on average from the time these goods arrived to their point of entry (e.g. port, airport) until the time these goods could be claimed from customs?

	**Days**
Average number of days to clear customs	d14
**Less than one day**	**1**
**Don't know (spontaneous)**	**-9**

**D.16**	At the present time, when this establishment receives delivery of its most important input, on average, how many days of inventory, measured in days of production, does this establishment keep?<br>**(INTERVIEWER: if respondent requires clarification, define days of inventory as stock on hand)**

	**Days**
Days of inventory of most important input	**d16**
**Don't know (spontaneous)**	**-9**

**D.30**	Using the response options on the card; To what degree is **Transport** an obstacle to the current operations of this establishment? **SHOW CARD 7**<br><br>Using the response options on the card; To what degree is **Customs and Trade Regulation** an obstacle to the current operations of this establishment? **SHOW CARD 7**

	No obstacle	Minor obstacle	Moderate obstacle	Major obstacle	Very Severe Obstacle	**Don't Know (spontaneous)**	**Does Not Apply (spontaneous)**
Transport **d30a**	0	1	2	3	4	**-9**	**-7**
Customs and trade regulations **d30b**	0	1	2	3	4	**-9**	**-7**

SERIAL NUMBER [ ]

E. DEGREE OF COMPETITION

**E.1**	In fiscal year **2011**, which of the following was the main market in which this establishment sold its main product? **SHOW CARD 8**

Local – main product sold mostly in same municipality where establishment is located	1	
National – main product sold mostly across the country where establishment is located	2	
International	3	*GO TO QUESTION E.6*
**Don't know (spontaneous)**	**-9**	*GO TO QUESTION E.6*
		**e1**

**E.2**	In fiscal year **2011**, for the main market in which this establishment sold its main product, how many competitors did this establishment's main product face?

Number of competitors	**e2b**
**Too many to count**	**-4**
**Don't know (spontaneous)**	**-9**

**E.6**	Does this establishment at present use technology licensed from a foreign-owned company, excluding office software?

Yes	1
No	2
**Don't know (spontaneous)**	**-9**
	**e6**

**E.11**	Does this establishment compete against unregistered or informal firms?

Yes	1
No	2
**Don't know (spontaneous)**	**-9**
	**e11**

**E.30**	Using the response options on the card; To what degree are **Practices of Competitors in the Informal Sector** an obstacle to the current operations of this establishment? **SHOW CARD 9**

	No obstacle	Minor obstacle	Moderate obstacle	Major obstacle	Very Severe Obstacle	**Do Not Know (spontaneous)**	**Does Not Apply (spontaneous)**
Practices of competitors in the informal sector **e30**	0	1	2	3	4	**-9**	**-7**

SERIAL NUMBER [ ]

F. CAPACITY

**F.1**	In fiscal year **2011**, what was this establishment's output produced as a proportion of the maximum output possible if using all the resources available (capacity utilization)?

	**Percent**
Capacity utilization	**f1** %
**Don't know (spontaneous)**	**-9**

**F.2**	*In fiscal year **2011**, how many hours per week did this establishment normally operate?* ***(INTERVIEWER: response must be 168 hours or less)***

	**Hours**
Typical hours of operation in a week	**f2**
**Don't know (spontaneous)**	**-9**

SERIAL NUMBER | |

G. LAND AND PERMITS

G.1	Of the land occupied by this establishment, what percent is: **SHOW CARD 10**

	Percent	Don't know (spontaneous)
Owned by this establishment	g1a %	-9
Rented or leased by this establishment	g1b %	-9
Other	g1c %	-9
	100%	

INTERVIEWER: CHECK THAT TOTAL SUMS TO 100%
UNLESS RESPONDENT DOES NOT KNOW, IF IT IS A FLOOR IN A BUILDING, WRITE -7

G.2	Over the last two years, did this establishment submit an application to obtain a construction-related permit?

Yes	1	
No	2	*GO TO QUESTION G.30*
**Don't know (spontaneous)**	**-9**	*GO TO QUESTION G.30*
		g2

G.3	In reference to that application for a construction-related permit, approximately how many days did it take to obtain it from the day of the application to the day the permit was granted?

	Days
Wait for a construction-related permit	g3
**Less than one day**	**1**
**Still in process**	**-6**
**Application denied**	**-5**
**Don't know (spontaneous)**	**-9**

G.4	In reference to that application for a construction-related permit, was an informal gift or payment expected or requested?

Yes	1	
No	2	
**Don't know (spontaneous)**	**-9**	
**REFUSE (spontaneous)**	**-8**	
		g4

G.30	Using the response options on the card; To what degree is **Access to Land** an obstacle to the current operations of this establishment? **SHOW CARD 11**

	No obstacle	Minor obstacle	Moderate obstacle	Major obstacle	Very Severe Obstacle	**Do Not Know (spontaneous)**	**Does Not Apply (spontaneous)**
Access to land g30a	0	1	2	3	4	**-9**	**-7**

SERIAL NUMBER [ ]

O. INNOVATION AND TECHNOLOGY

READ THE FOLLOWING TO THE RESPONDENT BEFORE PROCEEDING:
We now turn to another topic.

**CNO.1**	In the last three years, has this establishment introduced any new products or services?

Yes	1	
No	2	*GO TO QUESTION CNO.3*
**Don't know (spontaneous)**	**-9**	*GO TO QUESTION CNO.3*
	**CNo1**	

**CNO.2**	In fiscal year **2011**, what percent of this establishment's total annual sales was accounted for by products or services that were introduced in the last three years?

	**Percent**
Percent of annual sales accounted for by new products or services	**CNo2** %
**Don't know (spontaneous)**	**-9**
**Not applicable (spontaneous)**	**-7**

INTERVIEWER READ OUT THE FOLLOWING: Research and development (R&D) is defined as creative work undertaken on a systematic basis in order to increase the stock of knowledge. For example, laboratory research for a new chemical compound of paint would be research and development while market research surveys or internet surfing would not be research and development.

**CNO.3**	In the last three years, did this establishment spend on research and development activities within the establishment?

Yes	1	
No	2	*GO TO QUESTION CNO.5*
**Don't know (spontaneous)**	**-9**	*GO TO QUESTION CNO.5*
	**CNo3**	

**CNO.4**	Over the last three years, how much did this establishment spend on research and development activities performed within this establishment on average annually?

	**Yuan**
Expenditures on R&D performed within this establishment (include personal cost, materials and investment)	**CNo4**
**Don't know (spontaneous)**	**-9**
**Not applicable (spontaneous)**	**-7**

**CNO.5**	In the last three years, did this establishment spend on research and development activities contracted with other companies?

Yes	1	
No	2	***GO TO QUESTION CNO.7***
**Don't know (spontaneous)**	**-9**	***GO TO QUESTION CNO.7***
	**CNo5**	

**CNO.6**	Over the last three years, how much did this establishment spend on research and development activities contracted with other companies on average annually?

	**Yuan**
Expenditures on R&D contracted with other companies	**CNo6**
**Don't know (spontaneous)**	**-9**
**Not applicable (spontaneous)**	**-7**

**CNO.7**	Over the last three years, how much did this establishment spend in the following categories on average annually?

**INTERVIEWER: READ EACH OPTION ALOUD**	**Yuan**	**Don't Know (spontaneous)**	**NA (spontaneous)**
Computers and other information processing equipment (including printers, terminals, optical and magnetic reader, RFID, operating systems and software)	**CNo7a**	**-9**	**-7**
Non-IT related technologies (e.g., equipments needed for production/operations/manufacturing)	**CNo7b**	**-9**	**-7**

**CNO.8**	**Currently**, what percent of this establishment's workforce regularly **use** computers in their jobs?

	**Percent**
Percentage of workforce that uses computers	**CNo8 %**
**Don't know (spontaneous)**	**-9**
**Not applicable (spontaneous)**	**-7**

**CNO.9**	**Three years ago**, what percent of this establishment's workforce regularly **used** computers in their jobs?

	**Percent**
Percentage of workforce that used computers three years ago	**CNo9 %**
**Don't know (spontaneous)**	**-9**
**Not applicable (spontaneous)**	**-7**

SERIAL NUMBER | |

**CNO.10**	In fiscal year **2011**, what is the percentage of sales revenue generated through Internet?

	**Percent**
Percentage of sales revenue generated through Internet	**CNo10 %**
No sales generated through Internet	**0**
**Don't know (spontaneous)**	**-9**
**Not applicable (spontaneous)**	**-7**

**CNO.11**	To what extent are information and communication technologies (computers, internet, and software) used to support key business activities in each of the following business processes? **SHOW CARD 12**

	Never ***1***	***Rarely (once in a while) 2***	***Sometime (few times a month) 3***	***Frequently (few times a week) 4***	***All the time (Daily) 5***	**Don't Know (spontaneous)**	**Does Not Apply (spontaneous)**
Partner relations (suppliers, contractors, etc.) **CNo11a**	1	2	3	4	5	**-9**	**-7**
Product and service enhancement **CNo11b**	1	2	3	4	5	**-9**	**-7**
Production and operations **CNo11c**	1	2	3	4	5	**-9**	**-7**
Marketing and sales **CNo11d**	1	2	3	4	5	**-9**	**-7**
Customer relations **CNo11e**	1	2	3	4	5	**-9**	**-7**

**CNO.12**	Currently, what types of communication medium and technologies have been used for inter-organizational relationships and transactions?

**INTERVIEWER: READ EACH OPTION ALOUD**		**Yes**	**No**	**Don't Know (spontaneous)**	**NA (spontaneous)**
Phone and Fax	**CNo12a**	1	2	**-9**	**-7**
E-mail	**CNo12b**	1	2	**-9**	**-7**
EDI (Electronic Data Interexchange)	**CNo12c**	1	2	**-9**	**-7**
Online web-based systems (Internet-based)	**CNo12d**	1	2	**-9**	**-7**
Software, such as enterprise resource planning (ERP) systems, supply chain management (SCM) systems, and customer relationship management (CRM) systems	**CNo12e**	1	2	**-9**	**-7**

**CNO.13**	Currently, what type of information is shared with trading partners?

**INTERVIEWER: READ EACH OPTION ALOUD**		**Yes**	**No**	**Don't Know (spontaneous)**	**NA (spontaneous)**
Sharing demand forecast with raw material supplier	**CNo13a**	1	2	**-9**	**-7**
Sharing production and replenishment plans with clients	**CNo13b**	1	2	**-9**	**-7**
Sharing raw material inventory with raw material suppliers	**CNo13c**	1	2	**-9**	**-7**
Sharing finished goods inventory with clients	**CNo13d**	1	2	**-9**	**-7**

**CNO.14**	Over the last three years, what type of innovation activities has this establishment engaged in?

**INTERVIEWER: READ EACH OPTION ALOUD**	**Yes**	**No**	**Don't Know (spontaneous)**	**NA (spontaneous)**
Introduce new technology and equipment(s) for product or process improvements **CNo14a**	1	2	**-9**	**-7**
Introduce new quality control procedure in production or operations **CNo14b**	1	2	**-9**	**-7**
Introduce new managerial/administrative processes **CNo14c**	1	2	**-9**	**-7**
Provide technology training for staff **CNo14d**	1	2	**-9**	**-7**
Introduce new product or new service **CNo14e**	1	2	**-9**	**-7**
Add new features to existing products or services **CNo14f**	1	2	**-9**	**-7**
Take measures to reduce production cost **CNo14g**	1	2	**-9**	**-7**
Take actions to improve production flexibility **CNo14h**	1	2	**-9**	**-7**

INTERVEWER:
IF ALL ANSWERS FOR QUESTION CNO.14 ARE "NO" OR "DON'T KNOW", GO TO QUESTION CNO.18

**CNO.15**	To what extent are information and communication technologies (computers, internet, and software) **used** to support each innovation activity in the following list? **SHOW CARD 13**

	No use 1	***Some use 2***	***Heavy use 3***	**Don't Know (spontaneous)**	**Does Not Apply (spontaneous)**
Introduce new technology and equipment(s) for product or process improvements **CNo15a**	1	2	3	**-9**	**-7**
Introduce new quality control procedure in production or operations **CNo15b**	1	2	3	**-9**	**-7**
Introduce new managerial/administrative processes **CNo15c**	1	2	3	**-9**	**-7**
Provide technology training for staff **CNo15d**	1	2	3	**-9**	**-7**
Introduce new product or new service **CNo15e**	1	2	3	**-9**	**-7**
Add new features to existing products or services **CNo15f**	1	2	3	**-9**	**-7**
Take measures to reduce production cost **CNo15g**	1	2	3	**-9**	**-7**
Take actions to improve production flexibility **CNo15h**	1	2	3	**-9**	**-7**

**CNO.16**	In fiscal year **2011**, what percent of this establishment's annual production volume was associated with new or improved processes introduced over the last three years?

	**Percent**
Percentage of production volume associated with new/improved processes	**CNo16 %**
**Don't know (spontaneous)**	**-9**
**Not applicable**	**-7**

SERIAL NUMBER [ ]

**CNO.17A**	In what ways has this establishment introduced **new products or services**?

**INTERVIEWER: READ EACH OPTION ALOUD**		**Yes**	**No**	**Don't Know (spontaneous)**	**NA (spontaneous)**
Developed or adapted in house	CNo17a	1	2	-9	-7
Developed in cooperation with suppliers	CNo17b	1	2	-9	-7
Developed in cooperation with client firms	CNo17c	1	2	-9	-7
Introduced your own version of a product already supplied (by another firm)	CNo17d	1	2	-9	-7
Implemented idea from internal R&D	CNo17e	1	2	-9	-7
Implemented idea from an external source, e.g. consultants, universities and research institutions	CNo17f	1	2	-9	-7

**CNO.17B**	In what ways has this establishment introduced **new or improved process**?

**INTERVIEWER: READ EACH OPTION ALOUD**		**Yes**	**No**	**Don't Know (spontaneous)**	**NA (spontaneous)**
Developed or adapted in house	CNo17g	1	2	-9	-7
Developed in cooperation with suppliers	CNo17h	1	2	-9	-7
Developed in cooperation with client firms	CNo17i	1	2	-9	-7
Licensed technology or process from another firm	CNo17j	1	2	-9	-7
Implemented idea from internal R&D	CNo17k	1	2	-9	-7
Implemented idea from an external source, e.g. consultants, universities and research institutions	CNo17m	1	2	-9	-7

**CNO.18**	Does this establishment have more than one operating units (e.g., departments, branches, business units, or product groups)?

Yes	1	
No	2	*GO TO QUESTION I.1*
**Don't know (spontaneous)**	**-9**	*GO TO QUESTION I.1*
		CNo18

**CNO.19**	For regular business activities/operations, how many operating units (e.g., departments, branches, business units, or product groups) of this establishment use data and software supplied centrally by the establishment?

None	1	
Some	2	
All	3	
**Don't know (spontaneous)**	**-9**	
**Not applicable (spontaneous)**	**-7**	
		CNo19

**CNO.20**	For regular business activities and operations, how many operating units (e.g., departments, branches, business units, or product groups) of this establishment follow a set of standardized processes or procedures developed centrally by the establishment?

None	1
Some	2
All	3
**Don’t know (spontaneous)**	**-9**
**Not applicable (spontaneous)**	**-7**

CNo20

I. CRIME

READ THE FOLLOWING TO THE RESPONDENT BEFORE PROCEEDING:
**We now turn to another topic.**

**I.1**	In fiscal year **2011**, did this establishment pay for security, for example equipment, personnel, or professional security services?

Yes	1	
No	2	*GO TO QUESTION I.3*
**Don’t know (spontaneous)**	**-9**	*GO TO QUESTION I.3*
	**i1**	

**I.2**	In fiscal year **2011**, what percentage of this establishment’s total annual sales was paid for security, or what was the total annual cost of security?

	**Percent**
Percentage of total annual sales for security	**i2a** %
**Don’t know (spontaneous)**	**-9**

PROVIDE EITHER ONE OR THE OTHER, NOT BOTH

	**Yuan**
Total annual cost of security	**i2b**
**Don’t know (spontaneous)**	**-9**

**I.3**	In fiscal year **2011**, did this establishment experience losses as a result of theft, robbery, vandalism or arson on this establishment’s premises?

Yes	1	
No	2	*GO TO QUESTION I.30*
**Don’t know (spontaneous)**	**-9**	*GO TO QUESTION I.30*
	**i3**	

**I.4**	In fiscal year **2011**, what were the estimated losses as a result of theft, robbery, vandalism or arson that occurred on this establishment's premises either as a percentage of total annual sales or as total annual losses?

	**Percent**
Losses as percentage of total annual sales	i4a %
**Don't know (spontaneous)**	**-9**

PROVIDE EITHER ONE OR THE OTHER, NOT BOTH

	**Yuan**
Total annual value of losses	**i4b**
**Don't know (spontaneous)**	**-9**

**I.30**	Using the response options on the card; To what degree is **Crime, Theft and Disorder** an obstacle to the current operations of this establishment? **SHOW CARD 14**

	No obstacle	Minor obstacle	Moderate obstacle	Major Obstacle	Very Severe Obstacle	**Do Not Know (spontaneous)**	**Does Not Apply (spontaneous)**
Crime, theft and disorder **i30**	0	1	2	3	4	**-9**	**-7**

SERIAL NUMBER [ ]

K. FINANCE

READ THE FOLLOWING TO THE RESPONDENT BEFORE PROCEEDING:
I would like to ask you a few questions about how you finance the operations of this establishment.

**K.1**	In fiscal year **2011**, what percentage, as a proportion of the value of total annual purchases of material inputs or services were paid for after delivery?

	Percent	Don't know (spontaneous)
Purchased on credit?	k1c %	-9

**K.2**	In fiscal year **2011**, what percentage of this establishment's total annual sales of its goods or services were paid for after delivery?

	Percent	Don't know (spontaneous)
Purchased on credit?	k2c %	-9

**K.3**	Over fiscal year **2011**, please estimate the proportion of this establishment's working capital that was financed from each of the following sources? Working capital is used to pay for day to day operations.<br>**INTERVIEWER: SHOW CARD 15**

	Percent	Don't know (spontaneous)
Internal funds or retained earnings	k3a %	-9
Borrowed from banks(private and state-owned	k3bc %	-9
Borrowed from non-bank financial institutions which include microfinance institutions, credit cooperatives, credit unions, or finance companies	k3e %	-9
Purchases on credit from suppliers and advances from customers	k3f %	-9
Other, moneylenders, friends, relatives, etc.	k3hd %	-9
	100%	

INTERVIEWER: CHECK THAT TOTAL SUMS TO 100%
(UNLESS RESPONDENT DOES NOT KNOW)

**K.4**	In fiscal year **2011**, did this establishment purchase any fixed assets, such as machinery, vehicles, equipment, land or buildings?

Yes	1	
No	2	*GO TO QUESTION K.6*
**Don't know (spontaneous)**	-9	*GO TO QUESTION K.6*
	k4	

**N.5**	In fiscal year **2011**, how much did this establishment spend on purchases of:

	**Yuan**	**Don't know (spontaneous)**
Machinery, vehicles, and equipment (new or used)	**n5a**	**-9**
Land and buildings	**n5b**	**-9**

**K.5**	Over fiscal year **2011**, please estimate the proportion of this establishment's total purchase of fixed assets that was financed from each of the following sources:<br>**SHOW CARD 16**

	**Percent**	**Don't know (spontaneous)**
Internal funds or retained earnings	**k5a**%	**-9**
Owners' contribution or issued new equity shares	**k5i** %	**-9**
Borrowed from banks: private and state-owned	**k5bc**%	**-9**
Borrowed from non-bank financial institutions	**k5e** %	**-9**
Purchases on credit from suppliers and advances from customers	**k5f** %	**-9**
Other, moneylenders, friends, relatives, bonds, etc	**k5hdj**%	**-9**
	**100%**	

INTERVIEWER: CHECK THAT TOTAL SUMS TO 100%
(UNLESS RESPONDENT DOES NOT KNOW)

**K.6**	Now let's talk about the establishment's current situation. At this time, does this establishment have a checking or savings account?

Yes	1	
No	2	
**Don't know (spontaneous)**	**-9**	
		**k6**

**K.7**	At this time, does this establishment have an overdraft facility?

Yes	1	
No	2	
**Don't know (spontaneous)**	**-9**	
		**k7**

**K.8**	At this time, does this establishment have a line of credit or a loan from a financial institution?

Yes	1	
No	2	***GO TO QUESTION K.15d***
**Don't know (spontaneous)**	**-9**	***GO TO QUESTION K.15d***
	**k8**	

**K.9**	Referring to the most recent line of credit or loan, what type of financial institution granted this loan? **SHOW CARD 17**

Private commercial banks	1
State-owned banks or government agency	2
Non-bank financial institutions	3
Other	4
**Don't know (spontaneous)**	**-9**
	**k9**

**K.10**	Referring only to this most recent line of credit or loan, in what year was the most recent line of credit or loan approved?

	**Year**
Year most recent loan or line of credit approved	**k10**
**Don't know (spontaneous)**	**-9**

INTERVIEWER: PROVIDE FOUR DIGITS FOR YEAR

**K.11**	Referring only to this most recent loan or line of credit, what was its value at the time of approval?

	**Yuan**
Size of most recent loan or line of credit approved	**k11**
**REFUSE (spontaneous)**	**-8**
**Don't know (spontaneous)**	**-9**

**K.13**	Referring only to this most recent loan or line of credit, did the financing require collateral?

Yes	1	
No	2	***GO TO QUESTION K.15b***
**Don't know (spontaneous)**	**-9**	***GO TO QUESTION K.15b***
	**k13**	

**K.14**	Referring only to this most recent loan or line of credit, what type of collateral was required? **INTERVIEWER: READ OUT**

**Collateral**		**Yes**	**No**	**Don't know (spontaneous)**
Land, buildings under ownership of the establishment	**k14a**	1	2	**-9**
Machinery and equipment including movables	**k14b**	1	2	**-9**
Accounts receivable and inventories	**k14c**	1	2	**-9**
Personal assets of owner (house, etc.)	**k14d**	1	2	**-9**
Other forms of collateral not included in the categories above	**k14e**	1	2	**-9**

SERIAL NUMBER [ ]

**K.15**	Referring only to this most recent line of credit or loan, what was the approximate value of the collateral required?

	**Yuan**
Value of collateral	**k15a**
**Don't know (spontaneous)**	**-9**

**K.15b**	What is the total number of outstanding loans or lines of credit held by this establishment?

	**Number**
Total number of loans/lines of credit outstanding	**k15b**
**Don't know (spontaneous)**	**-9**

**K.15c**	What is the total value of outstanding loans or lines of credit held by this establishment?

	**Yuan**
Total value of loans/lines of credit outstanding	**k15c**
**Don't know (spontaneous)**	**-9**

**K.15d**	At this time, do you have any outstanding personal loans that are used to finance this establishment's business activities?

Yes	1	
No	2	
**Don't know (spontaneous)**	**-9**	
		**k15d**

**K.16**	Referring again to the last fiscal year **2011**, did this establishment apply for any loans or lines of credit?

Yes	1	***GO TO QUESTION K.20***
No	2	
**Don't know (spontaneous)**	**-9**	***GO TO QUESTION K.20***
		**k16**

**K.17**	What was the **main** reason why this establishment did not apply for any line of credit or loan? **SHOW CARD 18**

No need for a loan - establishment had sufficient capital	1
Application procedures were complex	2
Interest rates were not favorable	3
Collateral requirements were too high	4
Size of loan and maturity were insufficient	5
Did not think it would be approved	6
Other	7
**Don't know (spontaneous)**	**-9**
	**k17**

**K.20**	Referring only to this most recent application for a line of credit or loan, what was the outcome of that application?

Application was approved	1
Application was rejected	2
**Still in process**	**-6**
**Don't know (spontaneous)**	**-9**
	**k20a**

**K.21**	In fiscal year **2011**, did this establishment have its annual financial statements checked and certified by an external auditor?

Yes	1
No	2
**Don't know (spontaneous)**	**-9**
	**k21**

**K.30**	Using the response options on the card; To what degree is **Access to Finance** an obstacle to the current operations of this establishment? **SHOW CARD 19**

		No obstacle	Minor obstacle	Moderate obstacle	Major obstacle	Very Severe Obstacle	**Do Not Know (spontaneous)**	**Does Not Apply (spontaneous)**
Access to finance	**k30**	0	1	2	3	4	**-9**	**-7**

SERIAL NUMBER | |

J. BUSINESS-GOVERNMENT RELATIONS

READ THE FOLLOWING TO THE RESPONDENT BEFORE PROCEEDING:
The following questions assess how establishments, such as this one, deal with government officials and their agencies.

**J.1**	. Please tell me if you Strongly disagree, Tend to disagree, Tend to agree, or Strongly agree with the following statement. **SHOW CARD 20**

	Strongly disagree	Tend to disagree	Tend to agree	Strongly agree	**Don't know (spontaneous)**	**Does not apply (spontaneous)**
"The court system is fair, impartial and uncorrupted." **h7a**	1	2	3	4	**-9**	**-7**

**J.2**	In a typical week over the last year, what percentage of total senior management's time was spent on dealing with requirements imposed by government regulations?<br>[By senior management I mean managers, directors, and officers above direct supervisors of production or sales workers. Some examples of government regulations are taxes, customs, labor regulations, licensing and registration, including dealings with officials and completing forms]

	**Percent**
Senior management's time spent on dealing with regulations	j2 %
**No time was spent**	**0**
**Don't know (spontaneous)**	**-9**

**J.3**	Over the last year**,** was this establishment visited or inspected by tax officials?

Yes	1	
No	2	*GO TO QUESTION J.6a*
**Don't know (spontaneous)**	**-9**	*GO TO QUESTION J.6a*
		j3

**J.4**	Over the last year, how many times was this establishment either inspected by tax officials or required to meet with them?

	**Number**
Times inspected or met with tax officials	j4
**Don't know (spontaneous)**	**-9**

**J.5**	In any of these inspections or meetings was a gift or informal payment expected or requested?

Yes	1	
No	2	
**Don't know (spontaneous)**	**-9**	
**REFUSE (spontaneous)**	**-8**	
		j5

SERIAL NUMBER [ ]

**J.6a**	Over the last year, has this establishment secured or attempted to secure a government contract?

Yes	1	
No	2	*GO TO QUESTION J.7*
**Don't know (spontaneous)**	**-9**	*GO TO QUESTION J.7*
	j6a	

**J.6**	When establishments like this one do business with the government, what percent of the contract value would be typically paid in informal payments or gifts to secure the contract?

	**Percent**
Percent of the contract value paid as informal payments or gifts	**j6** %
**Don't know (spontaneous)**	**-9**
**Refusal (spontaneous)**	**-8**
**No payments**	**0**

**J.7**	It is said that establishments are sometimes required to make gifts or informal payments to public officials to "get things done" with regard to customs, taxes, licenses, regulations, services etc. On average, what percentage of total annual sales, or estimated total annual value, do establishments like this one pay in informal payments or gifts to public officials for this purpose?

	**Percent**
Percentage of total annual sales paid as informal payment	**j7a** %
**No payments or gifts are paid**	**0**
**Don't know (spontaneous)**	**-9**
**Refusal (spontaneous)**	**-8**

PROVIDE EITHER ONE OR THE OTHER, NOT BOTH

	**Yuan**
Total annual informal payment	**j7b**
**No payments or gifts are paid**	**0**
**Don't know (spontaneous)**	**-9**
**Refusal (spontaneous)**	**-8**

**J.10**	Over the last two years, did this establishment submit an application to obtain an import license?

Yes	1	
No	2	*GO TO QUESTION J.13*
**Don't know (spontaneous)**	**-9**	*GO TO QUESTION J.13*
	j10	

SERIAL NUMBER [ ]

**J.11**	Approximately how many days did it take to obtain this import license from the day of the application to the day it was granted?

	**Days**
Wait for import license	j11
**Less than one day**	**1**
**Still in process**	**-6**
**Application denied**	**-5**
**Don't know (spontaneous)**	**-9**

**J.12**	In reference to that application for an import license, was an informal gift or payment expected or requested?

Yes	1	
No	2	
**Don't know (spontaneous)**	**-9**	
**REFUSE (spontaneous)**	**-8**	
		j12

**J.13**	Over the last two years, did this establishment submit an application to obtain an operating license?

Yes	1	
No	2	***GO TO QUESTION J.30***
**Don't know (spontaneous)**	**-9**	***GO TO QUESTION J.30***
		j13

**J.14**	Approximately how many days did it take to obtain this operating license from the day of the application to the day it was granted?

	**Days**
Wait for operating license	j14
**Less than one day**	**1**
**Still in process**	**-6**
**Application denied**	**-5**
**Don't know (spontaneous)**	**-9**

**J.15**	In reference to that application for an operating license, was an informal gift or payment expected or requested?

Yes	1	
No	2	
**Don't know (spontaneous)**	**-9**	
**Refusal (spontaneous)**	**-8**	
		j15

SERIAL NUMBER [ ]

J.30	**Using the response options on the card; To what degree is/are [INSERT OPTION] an obstacle to the current operations of this establishment?** **SHOW CARD 21**

**ROTATE OPTIONS**		No obstacle	Minor obstacle	Moderate obstacle	Major obstacle	Very Severe Obstacle	**Do Not Know (spontaneous)**	**Does Not Apply (spontaneous)**
Tax rates	**j30a**	0	1	2	3	4	**-9**	**-7**
Tax administration	**j30b**	0	1	2	3	4	**-9**	**-7**
Business licensing and permits	**j30c**	0	1	2	3	4	**-9**	**-7**
Political instability	**j30e**	0	1	2	3	4	**-9**	**-7**
Corruption	**j30f**	0	1	2	3	4	**-9**	**-7**
Courts	**h30**	0	1	2	3	4	**-9**	**-7**

L. LABOR

READ THE FOLLOWING TO THE RESPONDENT BEFORE PROCEEDING:
Now I would like to ask you a few questions about this establishment's labor force.

**L.1**	At the end of fiscal year **2011,** how many permanent, full-time individuals worked in this establishment? Please include all employees and managers<br>(Permanent, full-time employees are defined as all paid employees that are contracted for a term of one or more fiscal years and/or have a guaranteed renewal of their employment contract and that work a full shift)<br>**(INTERVIEWER: include interviewee if applicable**).

	**Number**
Permanent, full-time workers end of last fiscal year	**l1**
**Don't know (spontaneous)**	**-9**

**L.2**	Three fiscal years ago, at the end of fiscal year **2009,** how many permanent, full-time individuals work in this establishment? Please include all employees and managers **(INTERVIEWER: include interviewee if applicable**).

	**Number**
Permanent, full-time workers three fiscal years ago	**l2**
**If establishment was not in business three years ago**	**-7**
**Don't know (spontaneous)**	**-9**

**L.3**	At the end of fiscal year **2011**, how many permanent, full-time individuals in this establishment were: **INTERVIEWER: READ EACH CATEGORY**

	**Number**	**Don't know (spontaneous)**
Production workers	**l3a**	**-9**
Non-production workers [e.g., managers, administration, sales]	**l3b**	**-9**

**L.4**	At the end of fiscal year **2011**, how many permanent, full-time individuals working in this establishment were: **INTERVIEWER: READ EACH CATEGORY**

	**Number**	**Don't know (spontaneous)**
Skilled production workers	**l4a**	**-9**
Unskilled production workers	**l4b**	**-9**

**L.5**	At the end of fiscal year **2011**, how many permanent full-time individuals working in this establishment in the following categories were female?

	**Number**	**Don't know (spontaneous)**
Female permanent full-time production workers	**l5a**	**-9**
Female permanent full-time non-production workers	**l5b**	**-9**

**L.6**	How many full-time temporary employees did this establishment employ throughout fiscal year **2011**?<br>**(INTERVIEWER:** Full-time, temporary workers are all paid short-term (i.e. for less than a fiscal year) employees with no guarantee of renewal of contract employment and work full days)

	**Number**
Full-time seasonal or temporary workers employed last fiscal year	**l6**
**Don't know (spontaneous)**	**-9**

IF 0, GO TO QUESTION L.9a

**L.6a**	How many female full-time temporary employees did this establishment employ throughout fiscal year **2011**?

	**Number**
Female full-time seasonal or temporary workers employed last fiscal year	**l6a**
**Don't know (spontaneous)**	**-9**

**L.8**	What was the average length of employment of all full-time temporary employees in fiscal year **2011**?

	**Months**
Average length full-time seasonal or temporary employment last fiscal year	**l8**
**Less than one month**	**1**
**Don't know (spontaneous)**	**-9**

**L.9a**	What is the average number of years of education of a typical permanent full-time production worker employed in this establishment?

READ OUT

	**Number**
Average number of years of education of typical production worker	**l9a**
Average number of years of education of typical Female production worker	**l9a2**
**Don't know (spontaneous)**	**-9**

**L.9b**	What is the percentage of full-time permanent workers who completed secondary school?

	**Percent**
Percentage of full time permanent workers who completed secondary school	**l9b**
**Don't know (spontaneous)**	**-9**

**L.10**	Over fiscal year **2011**, did this establishment have formal training programs for its permanent, full-time employees?

Yes	1	
No	2	*GO TO QUESTION l30a*
**Don't know (spontaneous)**	**-9**	*GO TO QUESTION l30a*
	**l10**	

**L.11**	Referring to the training programs run over fiscal year **2011**, what percentage of permanent, full-time employees of the following categories received formal training?

	**Percent**	**If no employees in a category were trained**	**Don't know (spontaneous)**
Production full-time permanent employees trained	**l11a** %	**0**	**-9**
Non-production full-time permanent employees trained	**l11b** %	**0**	**-9**

**L.30**	Using the response options on the card; To what degree are **Labor Regulations** an obstacle to the current operations of this establishment?<br><br>Using the response options on the card; To what degree is **an Inadequately Educated Workforce** an obstacle to the current operations of this establishment? **INTERVIEWER: SHOW CARD 21**

	No obstacle	Minor obstacle	Moderate obstacle	Major obstacle	Very Severe Obstacle	**Do Not Know (spontaneous)**	**Does Not Apply (spontaneous)**
Labor regulations **l30a**	0	1	2	3	4	**-9**	**-7**
Inadequately educated workforce **l30b**	0	1	2	3	4	**-9**	**-7**

SERIAL NUMBER | |

M. BUSINESS ENVIRONMENT

READ THE FOLLOWING TO THE RESPONDENT BEFORE PROCEEDING:

**M.1**	By looking at the card can you tell me which of the elements of the business environment included in the list, if any, currently represents the biggest obstacle faced by this establishment **SHOW CARD 22 Do NOT read out**

**1-Access to finance**
**2-Access to land**
**3-Business licensing and permits**
**4-Corruption**
**5-Courts**
**6-Crime, theft and disorder**
**7-Customs and trade regulations**
**8-Electricity**
**9-Inadequately educated workforce**
**10-Labor regulations**
**11-Political instability**
**12-Practices of competitors in the informal sector**
**13-Tax administration**
**14-Tax rates**
**15-Transport**

Biggest obstacle	**m1a**
**Don't know (spontaneous)**	**-9**
**Does not apply (spontaneous)**	**-7**

Rotation (option 1, 2 or 3)	**m1d**

SERIAL NUMBER [ ]

N. PERFORMANCE

READ THE FOLLOWING TO THE RESPONDENT BEFORE PROCEEDING:
Now, we would like to ask you a few questions about the financial results of this establishment. It is important that this information be as accurate as possible. The individual data are treated as confidential – the identity of your establishment will not be revealed at any point. Please provide the following information from the financial statements of this establishment.

**N.2**	For fiscal year **2011**, please provide the following information about this establishment by referring directly to your income statement: **SHOW CARD 23**

**READ OUT**	**Yuan**	**Don't know (spontaneous)**
Total annual cost of labor including wages, salaries, bonuses, social security payments	**n2a**	**-9**
Total annual cost of raw materials and intermediate goods used in production	**n2e**	**-9**
Total annual costs of fuel	**n2f**	**-9**
Total annual costs of electricity	**n2b**	**-9**
Total annual rental costs of vehicles, machinery, and equipment	**n2ra**	**-9**
Total annual rental costs of land, buildings	**n2rb**	**-9**
Other cost of production not included above	**n2j**	**-9**

**N.6**	Referring directly to your balance sheet, at the end of fiscal year **2011,** what was the net book value, that is the value of assets after depreciation, of the following:

	**Yuan**	**Don't know (spontaneous)**
Machinery, vehicles, and equipment	**n6a**	**-9**
Land and buildings	**n6b**	**-9**

**CNN.8**	In fiscal year **2011**, what were the firm's yearly average inventories?

	**Yuan**	**Don't know (spontaneous)**	**Not applicable (spontaneous)**
Finished goods inventory	**CNn8a**	**-9**	**-7**
Semi-finished goods inventory	**CNn8b**	**-9**	**-7**
Raw material inventory	**CNn8c**	**-9**	**-7**

**N.7**	Hypothetically, if this establishment were to purchase the assets it uses now, in their current condition, how much would they cost?

	**Yuan**	**Don't know (spontaneous)**
Machinery, vehicles, and equipment	**n7a**	**-9**
Land and buildings	**n7b**	**-9**

SERIAL NUMBER [ ]

A.15a Please complete the following information about the interviewee(s)

	Position in the firm	Years with the firm	Gender
Main respondent	a15a1ax	a15a2a	a15a3
Second respondent	a15a1bx	a15a2b	a15b3
Third respondent	a15a1cx	a15a2c	a15c3

THE SURVEY ENDS HERE

THANK YOU VERY MUCH FOR YOUR COOPERATION.

A.15 Time face-to-face interview ends:

Day (dd)	Month (mm)	Year (yyyy)	Hour (00 to 23)	Minutes (00 to 59)
a15d	a15m	a15y	a15h	a15min

INTERVIEWERS PLEASE ANSWER AT END OF THE INTERVIEW:

**A.16**	It is my perception that the responses to the questions regarding opinions and perceptions:

Truthful	1	
Somewhat truthful	2	
Not truthful	3	
		a16

**A.17**	The responses to the questions regarding figures (productivity and employment numbers):

Are taken directly from establishment records	1	
Are estimates computed with some precision	2	
Are arbitrary and unreliable numbers	3	
		a17

INTERVIEWER COMMENTS:

a17x

SERIAL NUMBER [ ]

(Problems occurred/extraordinary circumstances which could influence results)

SUPERVISORS PLEASE ANSWER:

**A.18**	This questionnaire was completed in:

One visit in face-to-face interview with one person	1	***STOP HERE***
One visit in face-to-face interview with different managers/staff	2	
Several visits	3	
		**a18**

**A.19**	If option 2 or 3 in **A.18,** estimate duration of the whole interview

**Hour**	**Minutes**
**a19h**	**a19m**
